# Biological functions and therapeutic potential of CKS2 in human cancer

**DOI:** 10.3389/fonc.2024.1424569

**Published:** 2024-08-12

**Authors:** Yueliang Lai, Ye Lin

**Affiliations:** ^1^ Department of Gastroenterology, Ganzhou People’s Hospital, Ganzhou, Jiangxi, China; ^2^ The Affiliated Ganzhou Hospital of Nanchang University, Ganzhou, Jiangxi, China

**Keywords:** cyclin-dependent kinase subunit 2 (CKS2), cancer, biomarker, targeted therapy, oncogene

## Abstract

The incidence of cancer is increasing worldwide and is the most common cause of death. Identification of novel cancer diagnostic and prognostic biomarkers is important for developing cancer treatment strategies and reducing mortality. Cyclin-dependent kinase subunit 2 (CKS2) is involved in cell cycle and proliferation processes, and based on these processes, CKS2 was identified as a cancer gene. CKS2 is expressed in a variety of tissues in the human body, but its abnormal expression is associated with cancer in a variety of systems. CKS2 is generally elevated in cancer, plays a role in almost all aspects of cancer biology (such as cell proliferation, invasion, metastasis, and drug resistance) through multiple mechanisms regulating certain important genes, and is associated with clinicopathological features of patients. In addition, CKS2 expression patterns are closely related to cancer type, stage and other clinical variables. Therefore, CKS2 is considered as a tool for cancer diagnosis and prognosis and may be a promising tumor biomarker and therapeutic target. This article reviews the biological function, mechanism of action and potential clinical significance of CKS2 in cancer, in order to provide a new theoretical basis for clinical molecular diagnosis, molecular targeted therapy and scientific research of cancer.

## 1 Introduction

Cancer is the biggest threat to public health in the world and the main cause of human death (1). With the increasingly serious environmental and aging problems, the incidence and mortality of malignant tumors are increasing year by year (2). According to the annual report of the World Health Organization (WHO), it is expected that in 2030, there will be 19.3 million new cancer cases and nearly 10 million cancer deaths worldwide (3). Oncogenes are important factors in human carcinogenesis, and the occurrence of cancer is caused by changes in oncogenes (gene mutation or deletion) caused by exogenous or endogenous factors, leading to the occurrence of malignant tumors (4, 5). Despite remarkable progress in multi-modal therapy such as surgery, chemotherapy and radiotherapy, the recurrence rate and mortality of tumors are still high and the prognosis is still far from ideal (6–8). In order to improve the survival rate and cure rate of cancer patients and reduce the mortality rate, gene therapy has been considered to combine with targeted therapy and chemotherapy to improve the survival rate and cure rate of cancer patients and reduce the mortality rate (9, 10). Therefore, it is very necessary to understand the mechanism of cancer formation and development as the focus of cancer research, and use this as the entry point to find new and effective biomarkers and therapeutic methods to accurately predict the prognosis of patients and/or the response to individualized therapy is of great significance for cancer prevention, treatment and prognosis (11).

Cyclin-dependent kinases (CKS) are small, highly conserved cyclin-dependent kinase (CDK) interacting proteins expressed in all eukaryotes (12). CKS proteins function as junctions that target cyclin-CDK complexes to CDK substrates (13). The CKS family consists of two subgene families of cyclin dependent kinase subunit 1 (CKS1) and CKS2 (14). Studies have reported that CKS proteins are often overexpressed in a variety of cancers and are involved in the maintenance of cancer cell phenotype and prognosis and outcome (15, 16).

**Figure 1:**
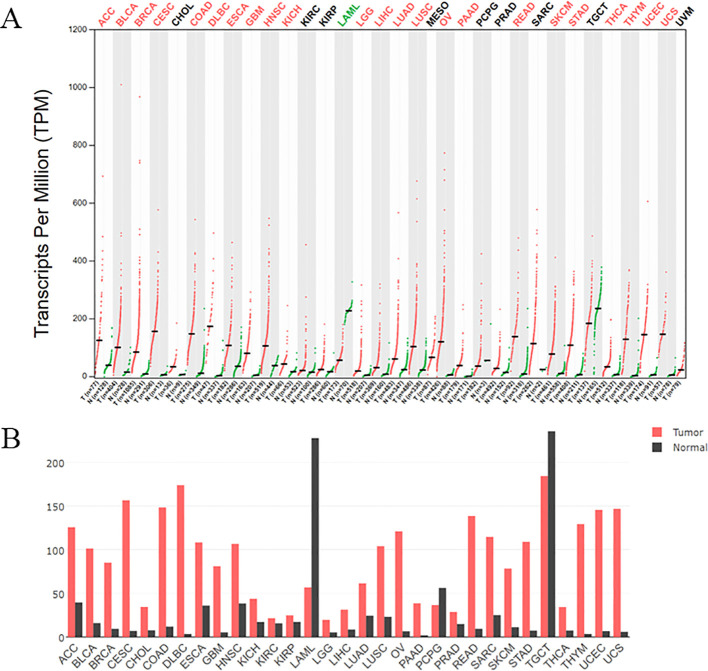
**(A)** The expression of CKS2 in various tumors was different from that in normal tissues. Log2FC: 1; Q - value: 0.01. (Data from GEPIA (Gene Expression Profiling Interactive Analysis)). N: Normal tissue, T: tumor tissue. **(B)** The gene expression profile across all tumor samples and paired normal tissues. The height of bar represents the median expression of certain tumor type or normal tissue.

**Figure 2:**
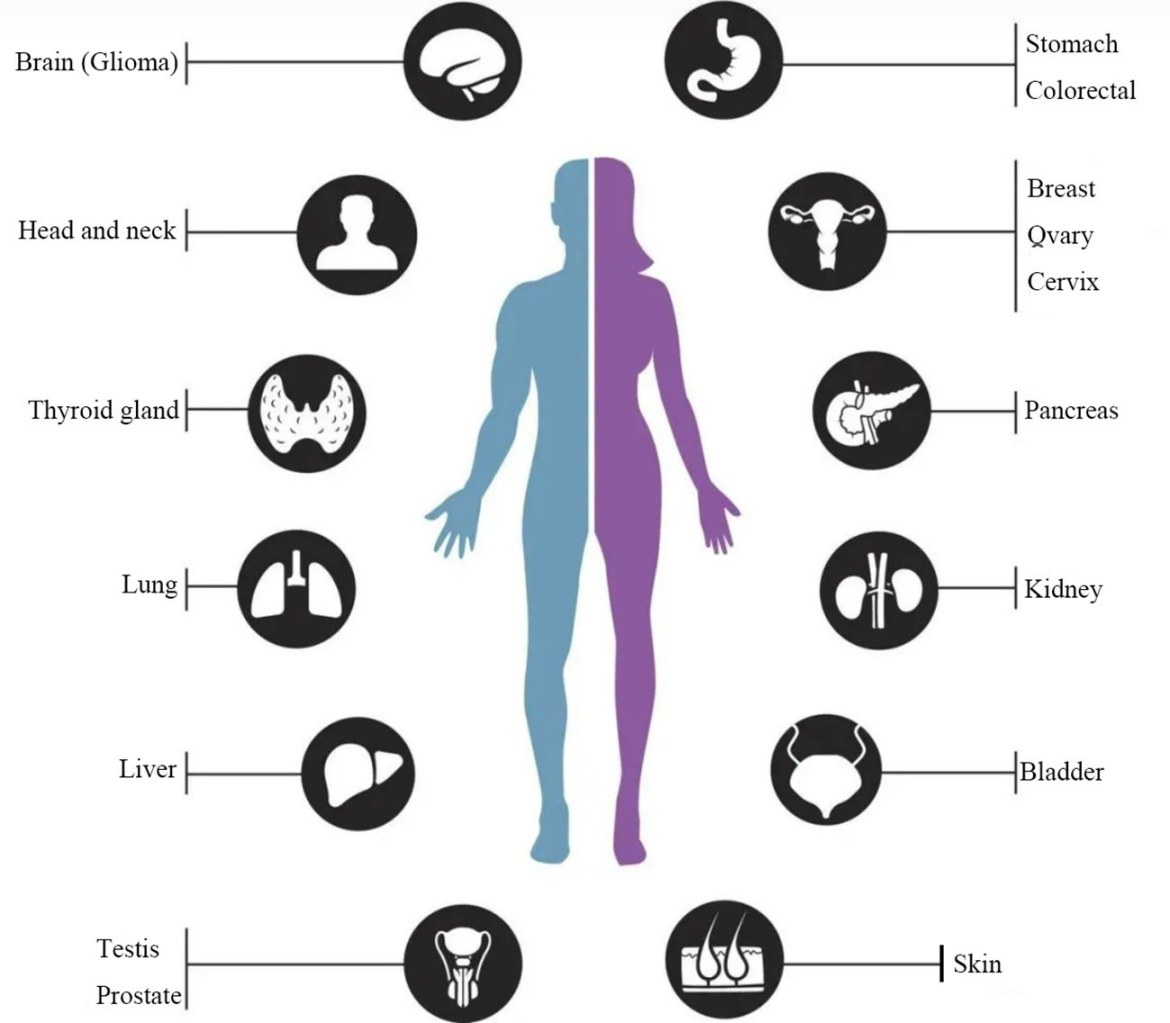
Dysregulation of CKS2 expression in multiple human cancers. CKS2 is up-regulated in gastric cancer, lung cancer, liver cancer, colorectal cancer, breast cancer, cervical cancer, glioma and other tumors, which can promote the proliferation, migration and invasion of cancer cells, inhibit cell apoptosis and predict poor prognosis.

Cyclin-dependent kinase subunit 2 (CKS2) is a member of the CKS protein family, and the CKS2 gene is located on chromosome 9q22 (14). CKS2 is expressed in various human systems, such as digestive tract, urogenital tract and respiratory system (17–19). And CDK binding plays a key role in somatic cell division and early embryonic development (20, 21). CKS2 binds to CDK1-cyclin B (CCNB) protein kinase, which is essential for mitosis and promotes the transition of the cell cycle from G2 to M (22, 23). At the same time, the binding of CKS2 to CDK2 may allow cells to override the S-phase checkpoint and continue DNA replication under replication stress (24). These unique functions based on CKS2 make it frequently associated with pathological changes in cancer. CKS2 is an important protein in intracellular signal transduction and cell division, and the expression pattern of CKS2 has been studied in a variety of cancers. In the TCGA database, CKS2 expression was significantly altered in multiple cancers compared to normal tissues (**Figure 1**). Previous studies have shown that abnormally high expression of CKS2 may act as an oncogene in the occurrence of various malignant tumors, and participate in carcinogenesis and tumor progression as well as maintenance of cancer cell phenotype (25) (**Figure 2**). In cell lines of multiple cancer types, overexpression of CKS2 has been shown to promote cell proliferation, metastasis, and migration and lead to poor prognosis (18). CKS2 knockdown can induce cell cycle arrest and apoptosis, and inhibit cell growth, migration and tumorigenesis in mice (19, 26). In addition, CKS2 can also promote tumor progression by inhibiting cancer cell apoptosis induced by chemotherapy drugs and reducing cell adhesion (27). This suggests that CKS2 can be used not only as a means of clinical diagnosis and prognosis of tumor patients, but also as a potential therapeutic target. In this review, we summarize the role, mechanism and potential clinical significance of CKS2 in cancer pathology. It provides a new theoretical basis for clinical molecular diagnosis, molecular targeted therapy and scientific research of cancer.

## 2 CKS2 and digestive system tumors

Digestive system malignancy is one of the most common tumors in the world (28) and the most common disease that causes human death (29, 30). Although the current diagnostic methods and treatment methods have made great progress compared with the previous ones, the early diagnosis and accurate treatment of cancer are still major challenges (31). Therefore, more in-depth studies are needed to further elucidate the underlying mechanisms of digestive system tumors in order to improve cure rates and reduce mortality. A large number of studies have shown that CKS2, as an oncogene, is highly expressed in digestive tract malignant tumors and is involved in the occurrence and development of various digestive system tumors. Such as stomach cancer, pancreatic cancer and liver cancer.

### 2.1 Liver cancer

Liver cancer is the most common malignant tumor with a high degree of malignancy and rapid development, and is the third leading cause of cancer-related death in the world (32). Despite advances in HCC prevention, diagnosis, and intervention in recent years, the treatment of this refractory disease remains unsatisfactory due to its poor prognosis and frequent recurrence (33). Recent studies have shown that CKS2 is associated with the occurrence and progression of liver cancer (34). Zhi et al. (35) confirmed the high expression of CKS2 in HCC at both mRNA and protein levels through multi-omics analysis, and the high expression of CKS2 in HCC can promote the progression of HCC by influencing the immune environment. While silenced CKS2 can inhibit proliferation and promote apoptosis by inhibiting the alternating expression of CDK2 and cleaved caspase-3 in HCC cells induced by EGFL7 overexpression (36). In addition, studies have also shown that CKS2 levels are significantly elevated in HCC tissues, and high expression of CKS2 may be significantly associated with the malignant phenotype of cancer cells and poor prognosis in patients with liver cancer. Through bioinformatics analysis, CKS2 may be associated with malignant phenotypes in HCC progression. Meanwhile, multivariate regression analysis showed that the overall survival rate of liver cancer patients with high CKS2 expression was significantly lower than that of patients with low CKS2 expression. Knockdown of CKS2 can significantly inhibit the proliferation, migration and invasion of HCC cells (37). These studies suggest that CKS2 may be a novel prognostic biomarker and potential target for liver cancer.

### 2.2 Gastric cancer

Gastric cancer (GC) is one of the most common malignant tumors in the world. Despite the continuous progress in the diagnosis and treatment of GC, the overall prognosis is still not ideal, with a 5-year survival rate of about 30% to 60% (38). Studies have shown that the expression of CKS2 in gastric cancer tissues is higher than that in adjacent normal tissues through TCGA database analysis (39), and the expression of CKS2 protein in gastric cancer tissues is closely related to the depth of tumor invasion and lymph node metastasis. At the same time, the results of bioinformatics analysis also confirmed that the high expression of CKS2 protein in GC tissues is closely related to the occurrence and development of GC, and can also be used as an independent factor to evaluate the prognosis of GC patients. In addition, Tanaka F et al. (19) found that CKS2 was significantly increased in gastric cancer tissues, and overexpression of CKS2 was associated with the proliferation, invasiveness and poor prognosis of tumor cells. The overall five-year survival rate of the group with high CKS2 expression was significantly lower than that of the group with low CKS2 expression. In addition, Kang et al. (40) found that CKS2 is significantly upregulated in gastric cancer, and that overexpression of CKS2 is correlated with tumor differentiation, tumor size, lymph nodes and metastasis.

### 2.3 Colorectal cancer

Colorectal cancer (CRC) is one of the most common human malignancies in the world and a major global healthcare burden (41). According to the statistical analysis of the American Association of Colorectal Surgeons (ACS), poor prognosis of CRC patients is related to primary tumor metastasis and spread (42). There is increasing evidence that changes in cell cycle regulation and signal transduction molecules contribute to tumor etiology and the pathogenesis of colorectal cancer (43). Data from the Oncomine database showed that CKS2 was significantly upregulated in CRC tissues compared to normal tissues. Yu et al. (44) also found that both mRNA and protein levels of CKS2 were up-regulated in CRC tissues. Overexpressed CKS2 contributes to the development and progression of CRC and is also significantly associated with tumor differentiation and lymph node metastasis. Multivariate Cox regression was also used to analyze the prognostic relationship between CKS2 and CRC, and the results showed that CKS2 was an independent prognostic factor for CRC. Knockdown of CKS2 can lead to decreased viability of CRC cells, increased apoptosis, cell cycle arrest, and decreased expression of cyclin (45). Studies have confirmed that the overexpression of CKS2 is closely related to the tumor aggressiveness and prognosis of various malignant tumors. Abnormal expression of CKS2 may contribute to the occurrence and progression of CRC, and CKS2 expression patterns may have diagnostic and prognostic value for CRC patients (46).

### 2.4 Other digestive system tumors

In addition to participating in the regulation of gastric cancer, liver cancer and colorectal cancer, CKS2 also acts as an oncogene in pancreatic cancer and esophageal cancer. Pancreatic cancer (PC) is the third leading cause of tumor-related death, accounting for an estimated 432,000 deaths annually. CKS2 expression levels were significantly elevated in PC tissues compared to adjacent normal tissues, and high CKS2 expression was associated with poor prognosis in PC patients. Knockdown of CKS2 can inhibit cell proliferation, induce cell cycle S phase, G2/M phase arrest and apoptosis *in vitro*, and reduce tumor growth *in vivo* (47). Esophageal cancer is one of the most aggressive cancers of the gastrointestinal tract and has a poor prognosis (48). Studies have shown that CKS2 mRNA expression in esophageal cancer tissues is significantly higher than that in normal tissues, and overexpression of CKS2 is correlated with depth of tumor invasion, lymphatic vessel invasion, clinical stage, distant metastasis and poor prognosis (49). These studies have shown that CKS2 is an important regulatory factor in digestive system tumors.

## 3 CKS2 and reproductive system tumors

Malignant tumors of urologic and genitourinary system are the malignant tumors that cause high death in males and females (50). Although breakthrough progress has been made in the diagnosis and treatment of malignant tumors of urinary and genitourinary systems in recent years (51, 52), the mortality and recurrence rate are still very high (53). This prompts us to urgently explore effective diagnostic biomarkers and therapeutic targets, which are of great significance for improving the prognosis of patients with malignant tumors of the urinary and genitourinary systems (9, 10, 54). Studies have found that CKS2 plays an important role in the development of bladder and prostate cancer, as well as breast cancer.

### 3.1 Breast cancer

Breast cancer (BC) is one of the most common malignant tumors in women worldwide, and its incidence has been increasing in recent years, with about 1.7 million newly diagnosed cases of breast cancer worldwide every year (50, 55). Because breast cancer is prone to invasion and metastasis, the clinical treatment of advanced breast cancer patients is often poor, which is the main cause of treatment failure and death of breast cancer patients (56). Therefore, early diagnosis and treatment can greatly improve the overall survival of breast cancer patients. Recent studies have shown that CKS2, as an oncogene, is significantly highly expressed in BC pathological tissues and cancer cells (57). Huang et al. (58) used Oncomine and the Cancer Genome Atlas database to detect CKS2 expression in BC, and found that CKS2 mRNA and protein levels were significantly increased in BC tissues, and high CKS2 expression was significantly correlated with the difference in overall survival, relapse-free survival and distant metastasis-free survival of BC patients. Inhibition of CKS2 can inhibit cell proliferation and invasion *in vitro* and reduce tumor growth *in vivo*. In addition, Wang et al. also found that CKS2 is significantly upregulated in breast cancer, and overexpression of CKS2 is associated with large tumor size, lack of progesterone receptor expression, poor tumor differentiation and reduced overall survival rate, and is an independent prognostic factor for breast cancer patients after radical resection (59).

### 3.2 Cervical cancer

Cervical cancer is one of the most common malignant tumors in gynecology, especially in postmenopausal women (60). According to statistics, 604,127 newly diagnosed cases and 341,831 deaths were reported in 2020 (3). Although great progress has been made in screening tests and treatment strategies, patients with cervical cancer often experience metastasis or recurrence and have a poor prognosis (61, 62). Therefore, the discovery of new effective biomarkers may be beneficial to improve the overall survival of cervical cancer patients. Studies have shown that CKS2 is overexpressed in cervical cancer as an oncogene and is related to the clinical progression of cervical cancer (63). It is also believed that the carcinogenic effect of CKS2 overexpression in cervical cancer is related to cell cycle and DNA replication (64). In addition, it has been suggested that MYC activation is related to the function of CKS2 in mtDNA replication. In tumors with high CKS2 expression, MYC transcriptional activity may lead to up-regulation of SSBP1. SSBP1 is a protein required to stabilize single-stranded DNA during mitochondrial DNA (mtDNA) replication (65). Jonsson M et al. (66) found that CKS2 forms a complex with positively related MYC target mitochondrial single strand DNA binding protein SSBP1 in the mitochondria of cervical tumor samples and HeLa and SiHa cervical cancer cell lines, thereby affecting patient prognosis. This suggests that CKS2 plays an important role in cervical cancer.

### 3.3 Prostate cancer

Prostate cancer is one of the malignancies affecting men and is the highest cause of cancer-related mortality in Western countries (67). Genomics studies have identified certain molecules that are important to the development of cancer, such as CKS2. The TCGA database and GSE21032 verification found that highly expressed CKS2 plays a crucial role in the occurrence, development, recurrence, metastasis and progression of prostate cancer (68). Lan et al. (26) found that CKS2 is highly expressed in prostate, and the highly expressed CKS2 is related to the malignant phenotype in the progression of prostate cancer, promoting proliferation, migration and invasion, while inhibition of CKS2 can reverse the above results.

## 4 CKS2 and other tumors

In addition to the cancers mentioned above, CKS2 is also highly expressed in other malignancies, such as lung cancer (69), glioma (70, 71) and thyroid cancer (72).

### 4.1 Lung cancer

Lung cancer is one of the most common malignancies worldwide and is the leading cause of cancer-related death (73). Lung cancer is divided into non-small cell lung cancer (NSCLC) and small cell lung cancer (74). As a cell cycle-associated protein, CKS2 plays an important role in tumor progression and prognosis. Replication protein A (RPA) is a single-stranded DNA binding protein that plays an important role in DNA replication, DNA recombination, cell cycle checkpoint and DNA repair (75). Replication protein A3 (RPA3) is an important subunit of the RPA protein complex and has been reported to play an important regulatory role in tumorigenesis (76). Chen et al. (77) found that RPA3 and CKS2 were highly expressed in lung adenocarcinoma cell lines, and silencing RPA3 could inhibit cancer cell viability, block cell cycle and promote cell apoptosis. There is an interaction between RPA3 and CKS2, and CKS2 overexpression can reverse the effects of RPA3 silencing on apoptosis and cell cycle. In the TCGA data, CKS2 expression level in lung adenocarcinoma was higher than that in normal tissues, and high CKS2 expression was associated with poor prognosis and malignant phenotype of lung adenocarcinoma, while silencing CKS2 expression significantly inhibited the proliferation and invasion of lung adenocarcinoma cells and promoted apoptosis (18). In addition, Feng et al. found that patients with high CKS2 expression in lung adenocarcinoma were more likely to relapse than those with low CKS2 expression, and overexpression of CKS2 would worsen the prognosis of patients with stage I-III invasive non-mucinous lung adenocarcinoma (78). In addition, CKS2 expression is also significantly up-regulated in NSCLC tissues and cells. Up-regulation of CKS2 can induce the growth and cell cycle progression of cancer cells, while knockdown of CKS2 can inhibit cell proliferation, induce cell cycle arrest, and increase the expression of P53, P21 and PTEN. Meanwhile, *in vivo* experiments in nude mice, knockdown CKS2 expression was also found to inhibit tumorigenesis *in vivo* animal models (79). It can be seen that CKS2 is an oncogene, and CKS2 can be used as a biomarker of lung cancer progression and prognosis. Glioma is the most typical malignant brain cancer.

### 4.2 Glioma

Glioma is characterized by rapid development, high degree of infiltration, difficult surgical resection, and high mortality (80). CKS2 is highly expressed in glioma compared to normal brain tissue. CKS2 acts as an oncogene in glioma to promote cell proliferation, invasion and epithelial-mesenchymal transformation pathway, while CKS2 knockdown can inhibit the proliferation, invasion and epithelial-mesenchymal transformation of glioma cell lines (81). In addition, CKS2 may be a candidate prognostic biomarker for glioma and may predict survival in glioma patients. Multivariate analysis of TCGA and CGGA data indicated that increased CKS2 expression was an independent risk factor for overall survival and prognosis in glioma patients. In addition, Kaplan-Meier survival analysis and Cox regression analysis showed that CKS2 was independently associated with poor prognosis in glioma patients (82).

### 4.3 Thyroid cancer

Thyroid cancer (PTC) is the most common endocrine system tumor, and surgical resection combined with radioactive iodine and levothyroxine therapy is the main treatment for PTC patients (83, 84). Although most PTC has a good prognosis, the rate of postoperative recurrence and lymphatic metastasis is high (85). Accumulating evidence suggests that CKS2 is an oncoprotein that promotes cancer tumorigenicity and progression, and that CKS2 levels in PTC correlate with the expression of its downstream genes, including cyclin B1 and CDK1. Knockdown of CKS2 expression using CKS2-siRNA in cancer cells can significantly inhibit cell proliferation, cell migration and invasion (86) (**Table 1**).

**Table 1:** The role and related mechanism of CKS2 in tumor.

Tumors	Expression of CKS2	Effect of high expression in tumor	Inhibition of CKS2 in tumor	Reference
Liver cancer	High expression	Promote proliferation, Migration and invasion	Inhibit proliferation, invasion and promote apoptosis	(37)
Gastric cancer	High expression	Promote proliferation and invasion	Inhibit proliferation and invasion	(19, 40)
Colon cancer	High expression	Promote proliferation, Migration and invasion	Inhibit proliferation, invasion, Migration and promote apoptosis	(44, 45)
Pancreatic cancer	High expression	Promote proliferation	Inhibit proliferation and promote apoptosis	(47)
Esophagus cancer	High expression	Promote migration and invasion	Inhibit migration and invasion	(49)
Breast Cancer	High expression	Promote proliferation, migration and low survival rate	Inhibit proliferation, migration and improve patient survival rate	(58, 59)
Cervical cancer	High expression	Promote proliferation and invasion	Inhibit proliferation and invasion	(64, 65)
Prostatic cancer	High expression	Promote proliferation, migration and invasion	Inhibit proliferation, migration and invasion	(26, 68)
Lung cancer	High expression	Promote proliferation and invasion	Inhibit proliferation, migration and promote apoptosis	(77–79)
Glioma	High expression	Promote proliferation and invasion	Inhibit proliferation and migration	(81, 82)
Thyroid cancer	High expression	Promote proliferation, migration and invasion	Inhibit proliferation, migration and invasion	(86)

## 5 Biological role of CKS2 in cancer

**Figure 3:**
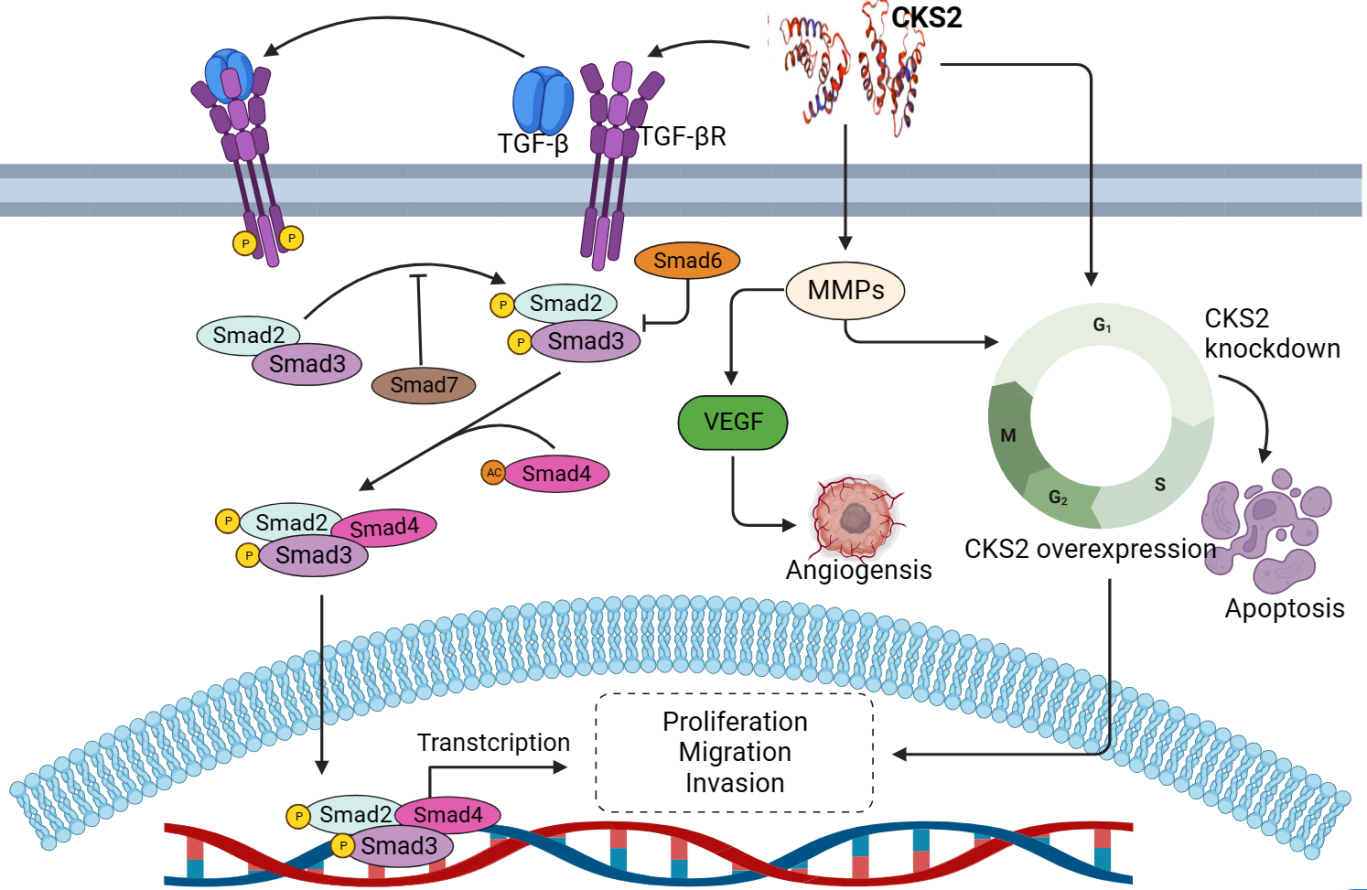
The role and mechanism of CKS2 in cancer.

The occurrence and development of cancer is a complex process, including excessive cell proliferation, cell resistance to death, invasion and metastasis (87). As an oncogene, CKS2 regulates the occurrence and development of various cancers. Abnormal expression of CKS2 mediates cell cycle regulation, tumor invasion, metastasis and apoptosis during the development of malignant tumors (**Figure 3**).

### 5.1 Proliferation

CKS2 has been shown to play an important role in the cell cycle, with up-regulation of CKS2 promoting cell proliferation and tumor formation in cell lines of multiple cancer types, while deletion or knockout of CKS2 leads to reduced cell proliferation, delayed DNA replication, and reduced clonal growth (88, 89). As downstream genes of CKS2, cdk1 and cyclin B1 are important players in the cell cycle. CKS2 binds to cdk1 by interacting with the catalytic subunit of cdk1, thereby affecting the cell cycle (22). In thyroid papillary carcinoma, CKS2 regulates the expression of cyclinB1 and cdk1, promotes cell growth, cell migration and invasion, and accelerates the G0/G1 phase, while knockdown CKS2 expression can reverse the above results (86). p53 protein is an important tumor suppressor and an important cellular regulator involved in cell cycle arrest and apoptosis. Trans-activated p21 cip1 induces cell arrest in the G0/G1 phase by binding to CDK1/cyclin B1 (90, 91). CKS2 is significantly upregulated in gastric cancer, and high levels of CKS2 inhibit the expression of tumor suppressor factors p53 and p21 cip1, thereby promoting increased cell growth, while transfection of cancer cells with CKS2-siRNA to inhibit CKS2 can promote the expression of tumor suppressor genes (P50 and P21) and inhibit cell proliferation (40). In addition, as an important protein of p53, the expression of PTEN is always related to the activity of P53 protein. In liver cancer, the high expression of CKS2 protein may reduce the level of PTEN protein in HCC cells and inhibit the activity of P53 protein, thus promoting the proliferation of HCC cells. Meantime, CKS2 can promote the proliferation of hepatocellular cancer cells by down-regulating the expression of phosphatase and tensin homologues (92). In addition, as an important molecule regulating cell cycle, CKS2 promotes cell cycle progression in tongue squamous cell carcinoma through CCNB protein kinase binding, while silencing CKS2 can inhibit cell growth and cell cycle progression of cancer cells by inducing G2/M phase arrest (93). At the same time, inhibition of CKS2 expression has also been found in CRC to inhibit cancer cell viability, reduce cyclin expression and lead to cell cycle arrest (44). In addition, CKS2 can promote tumor cell proliferation and inhibit apoptosis induced by common chemotherapy drugs. The mechanism by which CKS2 inhibits apoptosis may be through promoting phosphorylation of Akt and GSK-3β. Akt can inhibit apoptosis and promote cell survival through phosphorylation activation, and regulate glycogen synthesis through GSK-3β, thereby blocking GSK-3β-mediated phosphorylation and degradation of cyclin D to regulate cell cycle (27, 94). These researchs suggest that CKS2 plays an important role in the regulation of cell cycle and apoptosis of cancer cells, and inhibiting the expression of this gene may be beneficial to prevent the proliferation of cancer cells and promote apoptosis.

### 5.2 Migration and invasion

Cancer metastasis is the spread of cancer cells from the primary tumor to distant sites and is an important cause of cancer-related death. Metastasis involves a variety of molecular mechanisms, such as epithelial-mesenchymal transition (EMT) and tight junction proteins (95). EMT is thought to be activated during tumor metastasis and is associated with loss of cell adhesion, cell polarity, and acquisition of invasive properties (96). Alterations in the structure of tight junctions and increased leakage of tight junctions in cancer are associated with cancer progression (97). Abnormal expression of CKS2 has been found to promote the aggressive behavior of cancer cells. CKS2 promotes tumor metastasis by regulating the cell compact linking protein claudin1 (98). Upregulation of Claudin1 can lead to cellular changes, including loss of cell polarity, abnormal cell organization, and reduced cell differentiation (99). In colorectal cancer (CRC), CKS2 promotes cell invasion of CRC by up-regulating claudin1 expression (44). CKS2 overexpression induces nucleoplasmic translocation of smad 2/3 and upregulates p-TGF-βR1, p-samd2, p-smad3 and p-smad7. In addition, CKS2 activates the TGFβ/SMAD signaling pathway to promote EMT-like processes in gliomas, thereby inducing the aggressive and malignant phenotype of God cancer (epithelial-stromal transformation) (100). Bioinformatics analysis found that CKS2 is related to invasion and metastasis, and the overexpression of CKS2 contributes to the migration and invasion of thyroid cancer (86). In addition, CKS2 overexpression in hepatocellular carcinoma is significantly correlated with the aggressive clinical features and malignant behavior of HCC cells (37). Lymph node metastasis is extremely common for cancer (101), and the high expression of CKS2 in esophageal, gastric and cervical cancer tissues is positively correlated with the incidence of lymphatic invasion (40, 49, 63). These studies suggest that CKS2 plays an important role in tumor invasion and metastasis, suggesting that CKS2 may be a potential diagnostic biomarker and therapeutic target for tumors.

### 5.3 Others

In addition to regulating cell cycle and promoting cancer migration and invasion in human malignancies, CKS2 has also been found to be involved in the regulation of cancer angiogenesis and drug resistance. Angiogenesis (development of new blood vessels) is a key process in the growth and progression of tumor cells, and tumor progression and invasion are usually accompanied by the production of new blood vessels (102). HIF and vascular endothelial growth factor (VEGF) transcription factors are important angiogenic mediators (103), and in cancer, multiple factors can stimulate the transcription of various growth factors and extracellular matrix (ECM) remodelers to create vasculature (104). The high expression of CKS2 may be through the activation of metal matrix proteins (MMPs). CKS2 and MMPs can jointly accelerate the proliferation of endothelial cells, and at the same time activate VEGF, thus accelerating the degradation of basement membrane and endothelial cell migration (105, 106). Chemotherapy resistance is an important and serious problem for the success of chemotherapy, as well as an important factor affecting tumor prognosis (107). In clinical treatment, paclitaxel is often used as part of adjuvant chemotherapy for lung cancer. It can promote microtubule polymerization and inhibit depolymerization, causing the spindle to lose its normal function and cell mitosis to stop (108–110). In a study on lung adenocarcinoma, it was found that CKS2 expression affected the sensitivity of lung adenocarcinoma cells to carboplatin and paclitaxel, and the higher CKS2 expression, the higher the sensitivity of paclitaxel. It may be that CKS2 is related to microtubule binding, causing the spindle to lose its normal function and inhibiting cell division (78). In addition, studies also believe that CKS2 can be used as a biomarker of drug resistance to radiotherapy and chemotherapy of cervical cancer. In cervical cancer patients, the drug resistance of those with high CKS2 expression is significantly higher than that of those with low CKS2 expression, but the exact mechanism is still unclear (66).

## 6 Clinical significance of CKS2 in human tumors

### 6.1 CKS2 as a biomarker for cancer diagnosis and prognosis

Biomarkers with high specificity and sensitivity are of great significance for the diagnosis and prognosis of cancer (111). The unique biochemical characteristics of CKS2 make it a potential cancer biomarker. CKS2 has been identified as a potential prognostic marker and is overexpressed in a variety of cancers. CKS2 expression is abnormally elevated in gliomas accompanied by peripheral diffusion and infiltration, and CKS2 overexpression is associated with the proliferation, invasion and migration of glioma cells as well as the shortened survival time of glioma patients (100). At the same time, high expression of CKS2 in various cancers such as liver cancer (37), breast cancer (58) and prostate cancer (26) is associated with malignant phenotypes in cancer progression, such as promoting proliferation, migration and invasion, while inhibition of CKS2 can reverse these pathological processes. In addition, in gastric cancer, the expression of CKS2 in gastric cancer tissues is higher than that in adjacent normal tissues, and the expression of CKS2 protein in gastric cancer tissues is closely related to the depth of tumor invasion and lymph node metastasis (39). In addition, CKS2 mRNA expression in esophageal cancer tissues was significantly higher than that in normal tissues, and overexpression of CKS2 was associated with depth of tumor invasion, lymphatic vessel invasion, clinical stage, distant metastasis and poor prognosis (49). From these results, it can be inferred that CKS2 may play a role as a tumor oncogene. CKS2 may promote cancer invasion and may be a useful biomarker for predicting disease outcomes and the need for early preventive treatment.

### 6.2 CKS2 serves as a promising target for cancer therapy

In recent years, gene therapy has made great progress in improving the treatment of various genetic diseases and cancers due to its flexibility, high efficiency and reduction of off-target effects (112). Indeed, the same biochemical characteristics that make CKS2 a suitable biomarker may make it a promising therapeutic target. As previously mentioned, the biological function and clinical significance of CKS2 in cancer provide an opportunity for CKS2 to be a promising target for cancer therapy. Overexpression of CKS2 has been shown to promote cell proliferation in cell lines of multiple cancer types, while CKS2 knock-out induces cell cycle arrest and apoptosis, and inhibits cell growth, migration, and tumorigenesis in mice. In hepatocellular carcinoma, silencing CKS2 can significantly inhibit the proliferation, migration and invasion of cancer cells, induce the cell cycle to stop in G1 phase, and increase the apoptosis induced by common chemotherapy drugs (27). The Caspase protein family plays a key role in mediating apoptosis. Down-regulation of CKS2 in gastric cancer cells has been reported to decrease cell proliferation and increase caspase-3 activation and Bax expression (19). In cervical cancer, high CKS2 expression is associated with the presence of lymph node metastasis at diagnosis and with poor survival after chemoradiotherapy. Therefore, we speculate that CKS2 may be a new candidate potential target for cancer therapy, and inhibition of CKS2 may be a promising cancer treatment approach.

### 6.3 The prognostic value of CKS2 in cancer

In recent years, CKS2 has been widely recognized as a key regulator of tumors, and more and more studies have found that CKS2 gene expression increases in various tumorgenesis processes and is associated with poor prognosis (113). CKS2 expression is significantly upregulated in epithelial ovarian cancer (EOC), and high CKS2 expression is associated with late FIGO stage, histological grade, and shorter overall survival in patients with EOC. Studies have also found that knockdown of CKS2 inhibits proliferation, invasion and migration of EOC cells *in vitro*, and CKS2 can promote EMT progression by regulating EMT-related molecules (114). In lung cancer, expression is upregulated in lung cancer compared to adjacent normal tissues. The survival and disease-free survival rates of lung adenocarcinomas with higher CKS2 expression are significantly lower than those of lung adenocarcinomas with lower CKS2 expression (115). Meanwhile, overexpression of CKS2 in esophageal cancer is associated with advanced clinical stage and poor survival. Moreover, in esophageal cancer patients with the same TNM stage, the expression level of CKS2 in tissues of patients with good prognosis is significantly lower than that of patients with poor prognosis (49). CKS2 expression levels are also significantly up-regulated in patients with esophageal cancer (49), liver cancer (37) and adrenal cortical cancer (116), and abnormally high CKS2 expression is associated with poor prognosis of patients. Based on the above evidence, it is not difficult to find that CKS2 has an important prognostic value in human cancers.

## 7 Role of CKS2 as a target gene in cancer therapy

CKS2 can affect the expression of downstream genes, mainly cyclin A, cyclin B1 and CDK1. In some studies, multiple results have suggested that CKS2 is a direct target for specific genes. MicroRNA (miRNA) is a group of conserved small non-coding RNAs (ncRNAs) with an endogenous length of 17–25 nucleotides (117). Gene expression can be regulated by binding to the 3’ untranslated region (3’-UTR) of the target gene, promoting mRNA degradation or protein translation inhibition. MiRNAs are involved in many important physiological and pathological processes and are widely dysregulated in various cancers (118). Overexpression of miR-7 in thyroid cancer may induce G0/G1 stagnation and inhibit cell proliferation, migration and invasion by regulating the expression of CyclinB1 and CDK by targeting CKS2 (86). CKS2 has been identified as a target of miR-26a-5p, and its expression is negatively regulated by miR-26a-5p (119). miR-26a is involved in the occurrence and progression of various tumors, and the expression of miR-26a is down-regulated in laryngeal squamous cell carcinoma, while the expression of CKS2 is increased. Overexpression of miR-26a can inhibit cell proliferation, migration and invasion by targeting CKS2 (105). In addition to miRNAs, long non-coding RNAs (lncRNAs) also appear to be involved in the regulation of pathological processes targeting CKS2 in cancer. lncRNAs are ncRNAs with a length of more than 200 nucleotides that lack coding ability (120, 121), but lncRNAs can act as sponge for miRNAs to regulate the expression of target genes (122, 123). LINC00657 acts as a sponge for miR-26a-5p, and LINC00657 negatively mediates miR-26a-5p to regulate the growth of esophageal cancer cells. CKS2 has been observed as a direct target of miR-26a-5p, and its expression is negatively regulated by miR-26a-5p. Knockdown of LINC00657 can inhibit the expression of CKS2, thereby inhibiting the proliferation, migration and invasion of esophageal cancer cells and inducing cell apoptosis (119).

## 8 Limitations and challenges of CKS2 research in malignant tumors

Most existing preclinical trial reports suggest that CKS2 plays an important role in malignant tumors. Current studies on CKS2 and malignant tumors have some limitations, including lack of depth of study design, incomplete study subjects, and targeted CKS2 therapy for cancer. At a macro level, most of the current research designs on CKS2 in the field of malignant tumors are still at the stage of cell or rodent research, and corresponding clinical studies are lacking. At the micro level, current research has focused on the protein expression of CKS2, rather than the gene and single-cell level. In addition, in the targeting of CKS2 in cancer therapy, the current treatment of cancer targeting CKS2 mainly focuses on the non-coding RNA level. It is widely believed that mirnas target gene networks and regulate complex cellular processes, including development, cell differentiation, cell migration, and cell death (124). Although hybrid targeting is critical for endogenous miRNA function, the study of single gene function using RNAi technology depends on selective gene silencing and inhibition of off-target effects (OTE) (125). In addition, RNAi technology has other non-specific properties, including general toxicity due to the presence of toxic sequence motifs (126). There are a number of rules to follow when designing si/SHRnas that inhibit these non-specific effects, which might otherwise affect the study of single gene function (127). In addition, due to the complexity of the tumor microenvironment, it is difficult to control the progression and recurrence of cancer with a single traditional treatment. Therefore, the combination treatment strategy has gradually become an inevitable trend in cancer treatment (128). In order to overcome these limitations, researchers are exploring combination therapies that target multiple checkpoint molecules or combine immune checkpoint inhibitors with other treatments such as chemotherapy, radiation, or targeted therapies (129). Although specific inhibitors and combination therapy are beneficial to the treatment and prognosis of malignant tumors, there are relatively few studies on CKS2 at present. The current research on CKS2 in the field of malignant tumors is in the pre-clinical research stage such as cells or rodents, and the transformation of CKS2 inhibitors and combination therapy is also in the blank stage. Therefore, the conversion of CKS2 inhibitors and combination therapy needs and further research. In addition, drug resistance in chemotherapy is a major obstacle to the treatment of cancer and other diseases, and the development of drug resistance depends in part on the genetic instability, heterogeneity, rapid mutation, cytogenetic changes, and intratumoral diversity of tumor cells (130). These limitations also make targeting CKS2 challenging in cancer therapy. off-target effects and drug resistance should be limited in targeting CKS2 in cancer therapy, and delivery methods can be improved to address these challenges (131). Therefore, subsequent studies should focus on targeting tumor microenvironment (TME) soluble factors using enhanced absorption of nanotherapeutic drugs or appropriate methods based on specific receptor-nanocarrier interactions to achieve tumor targeted therapy (132).

## 9 Conclusion

Cyclin-dependent kinase subunit 2 (CKS2) is expressed in various human systems of CKS protein family members, such as digestive tract, urogenital tract and respiratory system. CKS2 is closely related to the occurrence and development of a variety of diseases, including cancer, and previous studies have found that CKS2 acts as an oncogene and is abnormally expressed in a variety of human malignancies. The up-regulation of CKS2 plays a crucial role in the development and prognosis of tumors. CKS2 can increase cyclin, cyclin A, cyclin B1 and CDK1, thereby promoting cancer cell proliferation. Deletion of CKS2 resulted in reduced cell proliferation, delayed DNA replication and reduced clonal growth. In addition, CKS2 may be a marker of cancer metastasis or poor prognosis. At the same time, abnormal CKS2 expression is often associated with poor clinical outcomes and thus can be used as a potential predictor of survival in cancer patients.

In summary, abnormal expression of CKS2 in a variety of human malignant tumors can lead to the occurrence and development of cancer, and plays a role in almost all biological aspects of tumor (proliferation, migration, invasion, and drug resistance). Therefore, CKS2 may be a potential biomarker for cancer diagnosis and prognosis, and may also be a target for cancer therapy.
